# Supplementary material for: A genome-wide association study of imaging-defined atherosclerosis
Source: Nat Commun. 2025 Mar 31;16:2266. doi: 10.1038/s41467-025-57457-7 (PMC11958696; doi:10.1038/s41467-025-57457-7)

Supplementary Information

A genome-wide association study of imaging-defined atherosclerosis

Anders Gummesson[a, b], Per Lundmark[c], Qiao Sen Chen[d], Elias Björnson[b], Koen F. Dekkers[c], Ulf Hammar[c], Martin Adiels[e], Yunzhang Wang[f], Therese Andersson[g], Göran Bergström[b, h], Carl-Johan Carlhäll[i, j, k], David Erlinge[l], Tomas Jernberg[f], Fredrik Landfors[g], Lars Lind[m], Maria Mannila[n], Olle Melander[o, p], Carlo Pirazzi[q], Johan Sundström[r, s], Carl Johan Östgren[i, k], Cecilia Gunnarsson[t], Marju Orho-Melander[o], Stefan Söderberg[g], Tove Fall[c], Bruna Gigante[d, u]

[a]Region Västra Götaland, Sahlgrenska University Hospital, Department of Clinical Genetics and Genomics, Gothenburg, Sweden
[b]Department of Molecular and Clinical Medicine, Institute of Medicine, Sahlgrenska Academy, University of Gothenburg, Gothenburg, Sweden
[c]Department of Medical Sciences, Molecular Epidemiology, Uppsala University, Uppsala, Sweden
[d]Division of Cardiology, Department of Medicine, Karolinska Institutet, Stockholm, Sweden
[e]School of Public Health and Community Medicine, Institute of Medicine, University of Gothenburg, Gothenburg, Sweden
[f]Department of Clinical Sciences, Danderyd University Hospital, Karolinska Institutet, Stockholm, Sweden
[g]Department of Public Medicine and Clinical Health, Umeå University, Umeå, Sweden
[h]Region Västra Götaland, Sahlgrenska University Hospital, Department of Clinical Physiology, Gothenburg, Sweden
[i]Center for Medical Image Science and Visualization (CMIV), Linköping University, Linköping, Sweden
[j]Department of Clinical Physiology in Linköping, Linköping University, Linköping, Sweden
[k]Department of Health, Medicine and Caring Sciences, Linköping University, Linköping, Sweden
[l]Department of Clinical Sciences Lund, Cardiology, Lund University, Sweden
[m]Department of Medical Sciences, Clinical Epidemiology, Uppsala University, Uppsala, Sweden
[n]Department of Cardiology, Karolinska University Hospital, Stockholm, Sweden
[o]Department of Clinical Sciences in Malmö, Lund University, Malmö, Sweden
[p]Department of Emergency and Internal Medicine, Skåne University Hospital, Malmö, Sweden
[q]Region Västra Götaland, Sahlgrenska University Hospital, Department of Cardiology, Gothenburg, Sweden
[r]Department of Medical Sciences, Uppsala University, Uppsala, Sweden
[s]The George Institute for Global Health, University of New South Wales, Sydney, Australia
[t]Department of Biomedical and Clinical Sciences, Division of Clinical Genetics, Linköping University, Linköping, Sweden
[u]Department of Cardiology, Karolinska University Hospital, Stockholm, Sweden

Abbreviations:

CACS, coronary artery calcification score; CarPlaq, carotid plaque; CCTA, coronary computed tomography angiography, CT, computed tomography; eQTLs, expression quantitative trait loci; GWAS, genome-wide association study; LD, linkage disequilibrium, SCAPIS, Swedish CArdioPulmonary bioImage Study; SD, standard deviation; SIS, segment involvement score; SNP, single nucleotide polymorphism; SuSiE, Sum of Single Effects.

Supplementary Table S1. Characteristics of the SCAPIS study participants of European ancestry with imputed genotypes included in the present study.

Numbers are presented as mean (SD) or n (%).

n	27010
Males	13110 (48.5%)
Age, years	57.6 (4.4)
Current smoker	3156 (12.1%)
Heredity for MI	1822 (6.9%)
**Body composition**	
Body mass index, kg/m^2	26.9 (4.5)
Waist circumference, cm	94.4 (13.0)
**Blood pressure**	
Hypertension diagnosis	6012 (23.0%)
Systolic blood pressure, mmHg	126.2 (17.0)
Diastolic blood pressure. mmHg	77.7 (10.6)
**Glycemic status**	
Diabetes diagnosis	1901 (7.0%)
Glucose, mmol/L	5.76 (1.10)
Haemoglobin A1c, mmol/mol	36.4 (6.2)
**Lipid status**	
Lipid-lowering medication	2059 (7.9%)
Low density lipoprotein cholesterol, mmol/L	3.45 (0.96)
High density lipoprotein cholesterol, mmol/L	1.64 (0.50)
Triglycerides, mmol/L	1.23 (0.83)
Lipoprotein(a), mg/dL	0.22 (0.27)
**Imaging-defined atherosclerosis**	
Any coronary plaque on CCTA (SIS > 0)	10589 (42.7%)
SIS 1-3	7476 (30.1%)
SIS 4-6	2221 (9.0%)
SIS 7-11	892 (3.6%)
At least one coronary vessel with >50% stenosis	1413 (5.7%)
Any coronary calcification on CT (CACS > 0)	10645 (40.9%)
CACS 1-100	7460 (28.7%)
CACS 101-400	2164 (8.3%)
CACS >400	1021 (3.9%)
Any carotid plaque on ultrasound (CarPlaq > 0)	14813 (55.3%)
Unilateral (CarPlaq = 1)	7944 (29.6%)
Bilateral (CarPlaq = 2)	6869 (25.6%)

Supplementary Table S2. Extended gene annotations for all significant independent SNPs associated with either SIS, CACS or CarPlaq.

Annotation from Open Targets (https://www.opentargets.org/) refers to the gene with the highest score for being functionally implicated by each variant. GTEx expression quantitative trait loci (eQTL) are those significant in coronary, aortic or tibial artery tissue in the GTEx database version 8 (https://www.gtexportal.org/home/), with maximum three shown (lowest p-value, 2-sided). STARNET eQTLs are those significant in atherosclerotic aortic root (http://starnet.mssm.edu/), with maximum three shown (lowest p-value, 2-sided).

SNP	Gene(s)	Consequence	Nearest protein-coding gene	Open Targets gene	GTEx eQTL (artery)	STARNET eQTL (atherosclerotic aortic root)
rs17127656	LEPR	Intronic	LEPR	LEPR		
rs1909197	MIA3	Intronic	MIA3	MIA3	MIA3, AIDA	TAF1A
rs78049276	EDNRA	Intronic	EDNRA	EDNRA		EDNRA
rs12190315	LINC01600	ncRNA_intronic	MYLK4	MYLK4	LINC01600	
rs9349379	PHACTR1	Intronic	PHACTR1	PHACTR1	PHACTR1, GFOD1, LOC100130357	PHACTR1, GFOD1
rs9268807	HLA-DRA, HLA-DRB5	Intergenic	HLA-DRA	HLA-DRB5	HLA-DRB5, HLA-DQB2, HLA-DQA1	HLA-DRB6
rs1578654	LINC02542	ncRNA_intronic	IBTK	TENT5A		
rs74617384	LPA	Intronic	LPA	SLC22A3		
rs10455872	LPA	Intronic	LPA	SLC22A3		
rs2315065	LPA, PLG	Intergenic	PLG	PLG		
rs186696265	LPA, PLG	Intergenic	PLG	PLG		
rs2074633	HDAC9	UTR3	HDAC9	TWIST1	TWIST1	TWIST1
rs73225858	BMP1	Intronic	BMP1	BMP1	BMP1, PHYHIP	BMP1, POLR3D, PHYHIP
rs2594811	MRPL15, RNU105C	Intergenic	MRPL15			
rs28601761	TRIB1, LINC00861	Intergenic	TRIB1	TRIB1		
rs1537370	CDKN2B-AS1	ncRNA_intronic	CDKN2B	CDKN2A		CDKN2B-AS1, CDKN2B
rs10738606	CDKN2B-AS1	ncRNA_intronic	CDKN2B	CDKN2A		CDKN2B-AS1, CDKN2B
rs4977575	CDKN2B-AS1, DMRTA1	Intergenic	CDKN2B	CDKN2B		CDKN2B-AS1, CDKN2B
rs3891689	ASTN2	Intronic	ASTN2	TRIM32		
rs631414	LINC00841, C10orf142	Intergenic	CXCL12	CXCL12		
rs2000660	LOC105370361, COL4A1	Intergenic	COL4A1	COL4A2		
rs11617955	COL4A1	Intronic	COL4A1	COL4A1		
rs9515203	COL4A2	Intronic	COL4A2	COL4A2		IRS2
rs4243085	ADAMTS7	Intronic	ADAMTS7	ADAMTS7	ADAMTS7, ADAMTS7P3	ADAMTS7
rs7198873	CFDP1, TMEM170A	Intergenic	TMEM170A	CFDP1	BCAR1	BCAR1, TMEM170A
rs423984	FOXL1, LINC02189	Intergenic	FOXL1	MTHFSD	RP11-58A18.2, RP11-58A18.1	LINC02188
rs2088140	IGF2BP1	Intronic	IGF2BP1	ATP5MC1		HOXB-AS1
rs4423524	KANK2	UTR3	KANK2			
rs429358	APOE	Exonic	APOE	APOE		
rs7412	APOE	Exonic	APOE	APOE		PPP1R13L
rs9305545	LINC00310, KCNE2	Intergenic	MRPS6	MRPS6	MRPS6, LINC00310, SLC5A3	SLC5A3, MRPS6, LINC00310
rs9980618	LINC00310, KCNE2	Intergenic	MRPS6	MRPS6	MRPS6, LINC00310, SLC5A3	SLC5A3, MRPS6, LINC00310

Supplementary Table S3. Mendelian randomization results on the causal relationship between SIS and MI, including IVW (main analysis) and MR Egger.

The analysis was based on the 20 significant independent SNPs for SIS in SCAPIS (n=24,811). Data on genetic associations with MI was obtained from UK Biobank (n=487,202). Statistical test for exposure-outcome relationship refer to the built-in test for each respective model in R-package MendelianRandomization. P-values are 2-sided and without adjustments for multiple comparisons

**Method**	**Odds Ratio**	**95% CI**		**P-value**
Simple median	1.77	1.59	1.97	4.84E-25
Weighted median	1.85	1.69	2.03	5.16E-39
Penalized weighted median	1.85	1.69	2.04	8.02E-38
IVW	1.83	1.59	2.1	2.65E-17
Penalized IVW	1.82	1.73	1.92	6.52E-108
Robust IVW	1.8	1.57	2.06	5.26E-17
Penalized robust IVW	1.84	1.75	1.94	1.84E-116
MR-Egger	2.52	1.89	3.37	3.00E-10
(intercept)	0.952	0.916	0.99	0.015
Penalized MR-Egger	2.29	1.8	2.92	1.93E-11
(intercept)	0.963	0.936	0.991	0.010
Robust MR-Egger	2.44	1.7	3.52	1.53E-06
(intercept)	0.956	0.914	1	0.048
Penalized robust MR-Egger	2.23	1.78	2.8	3.60E-12
(intercept)	0.967	0.936	1	0.049

Supplementary Figure S1. Flowchart defining the GWAS population.

The grey box depicts the population described in Supplementary Table S1. SCAPIS, Swedish CArdioPulmonary bioImage Study; SD, standard deviation; GWAS, genome-wide association study; SIS, segment involvement score; CACS, coronary artery calcification score; CarPlaq, carotid plaque. Age refers to mean ±SD.

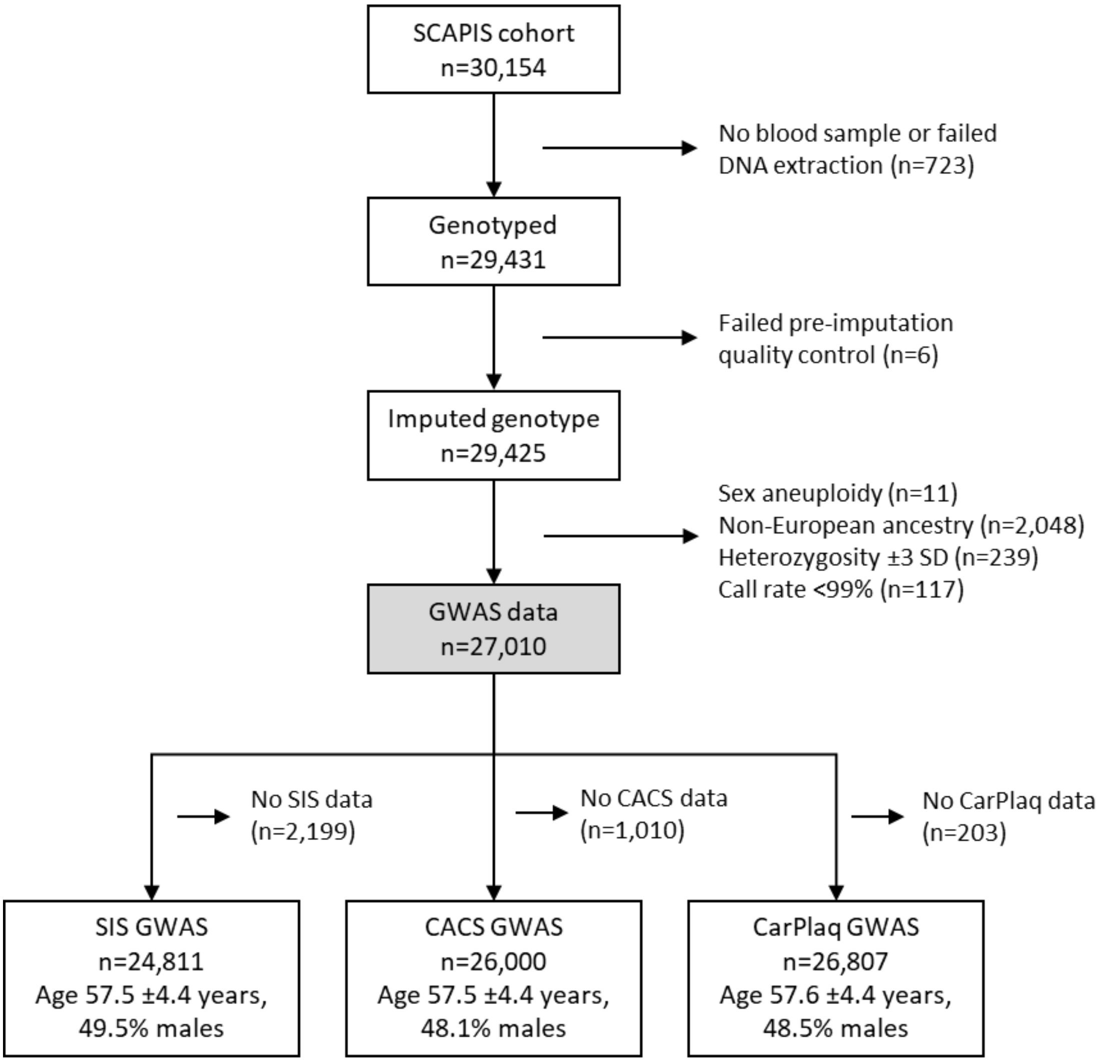

Supplementary Figure S2. Beta coefficients in the genetic associations with the segment involvement score (SIS) compared to plaque localization and severity.

The data are derived from GWAS of 24,811 SCAPIS participants. R^2 values are derived from linear regression. Left anterior descending artery include segments 5, 6, 7, 9 and 17; Left circumflex artery includes segments 11, 12 and 13; Right coronary artery includes segments 1, 2 and 3. Proximal segments include 1, 5, 6 and 11; Distal segments include 2, 3, 7, 9, 12, 13 and 17. Any segment with >50% stenosis was used to define severe atherosclerosis in the bottom-right panel. Error bars represent standard errors. Source data are provided as a Source Data file.

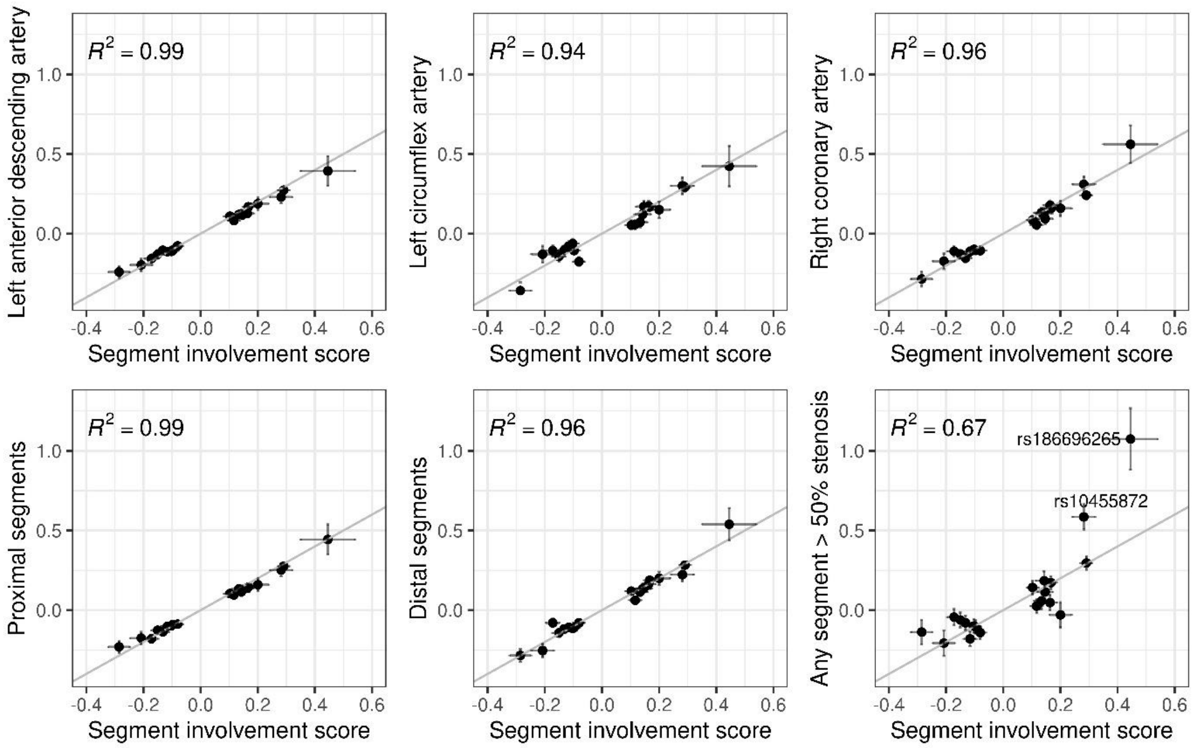

Supplementary Figure S3. Scatter plot of the multivariant colocalization between SIS and CarPlaq for rs10455872.

Genetic variants within a window of 250kb in the locus near rs10455872 were fine-mapped for each trait using SuSiE. In the scatter plot, one set of genetically colocalized SNPs between CarPlaq (blue) and SIS (red) are identified as triangles marked by black circles. Enlarged points represent fine-mapped SNPs for each trait. Below the scatter plot, the middle panel displays a line plot showing the genes and region associated with each SNP. At the bottom, the triangular heatmap shows the LD structure for the surrounding region. Source data are provided as a Source Data file.

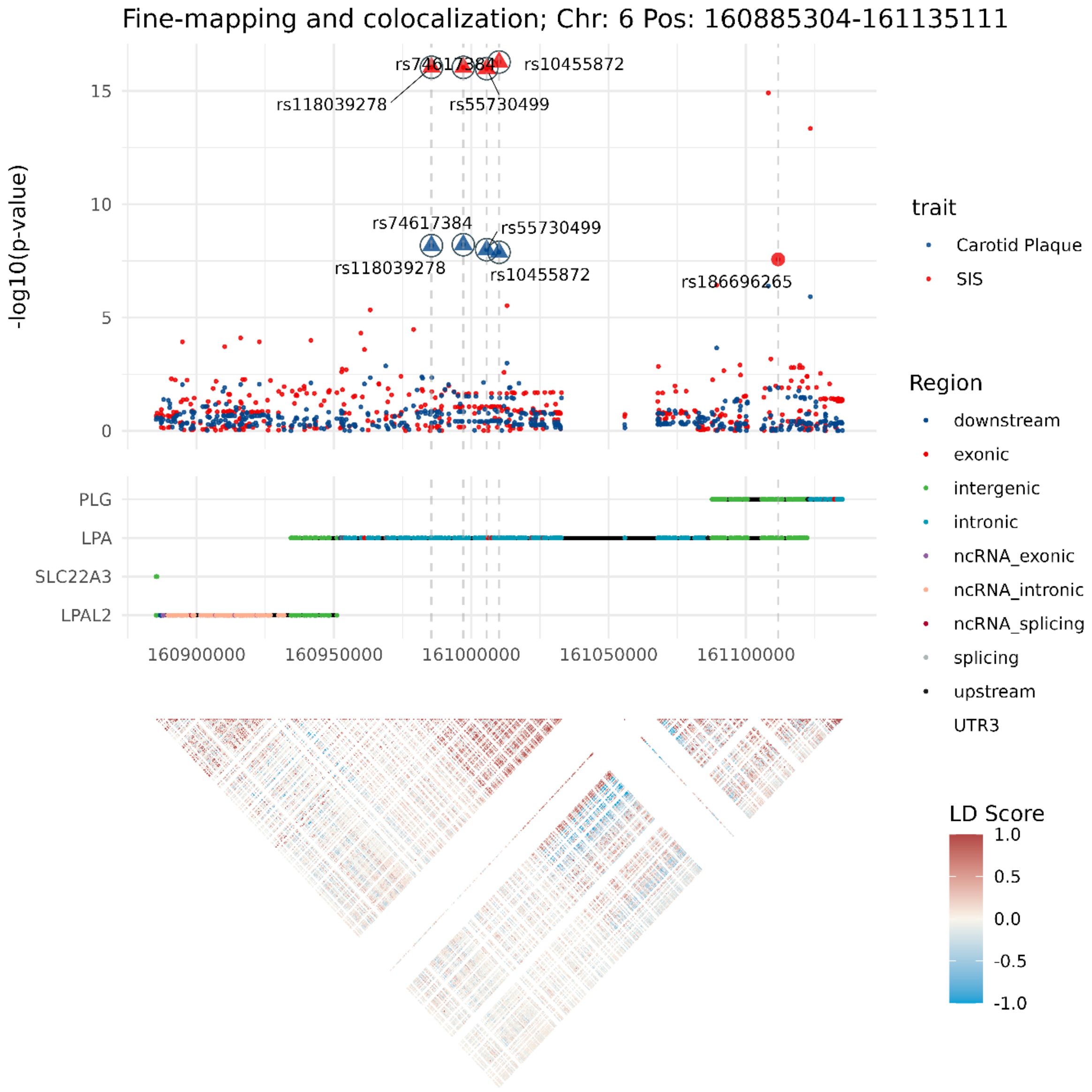

Supplementary Figure S4. Scatter plot of the multivariant colocalization between SIS and CarPlaq for rs186696265.

Genetic variants within a window of 250kb in the locus near rs186696265 were fine-mapped for each trait using SuSiE. In the scatter plot, one set of genetically colocalized SNPs between CarPlaq (blue) and SIS (red) are identified as triangles marked by black circles. Enlarged points represent fine-mapped SNPs for each trait. Below the scatter plot, the middle panel displays a line plot showing the genes and region associated with each SNP. At the bottom, the triangular heatmap shows the LD structure for the surrounding region. Source data are provided as a Source Data file.

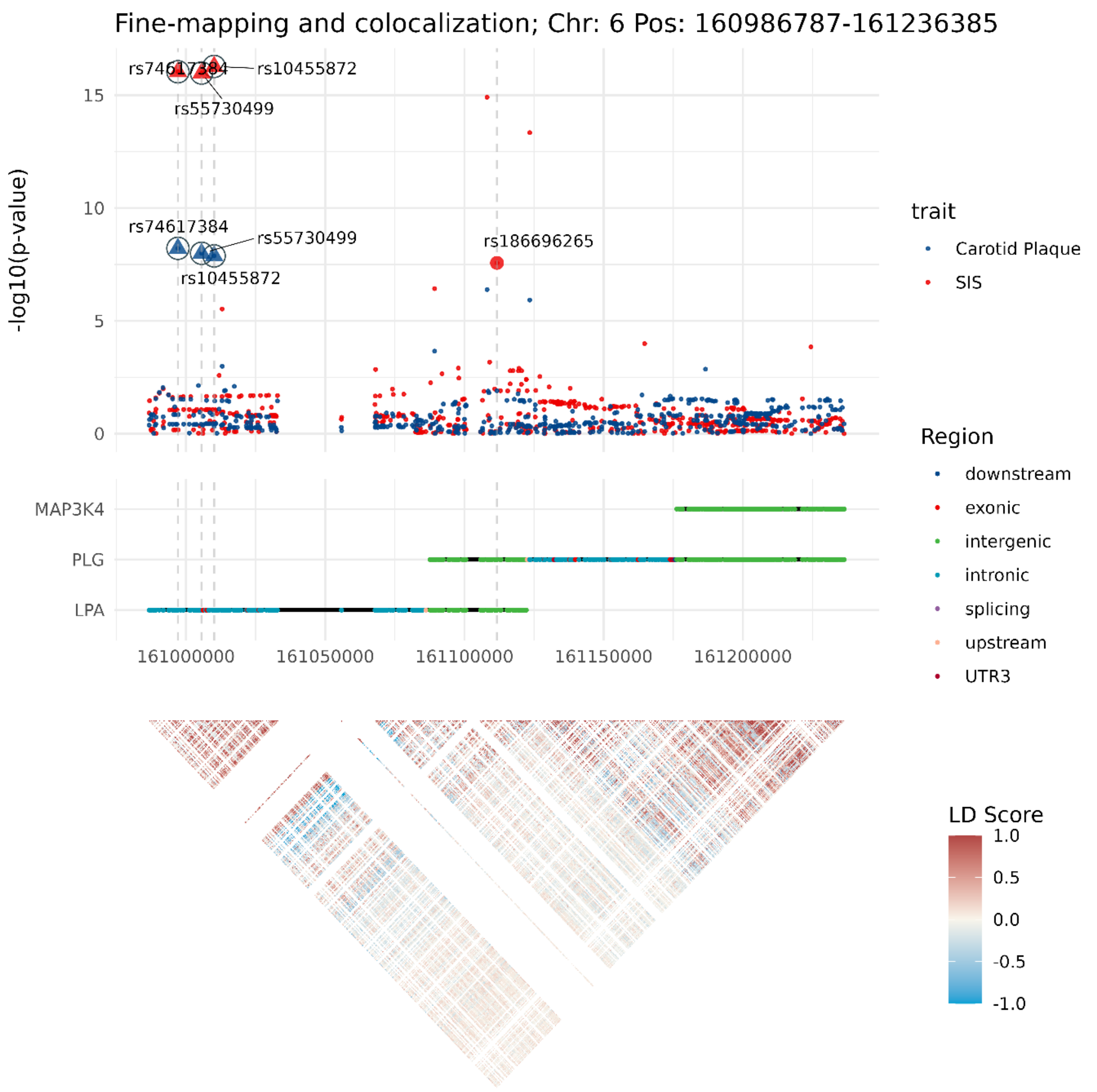

Supplementary Figure S5. Scatter plot of the multivariant colocalization between SIS and CarPlaq for rs28601761.

Genetic variants within a window of 250kb in the locus near rs28601761 were fine-mapped for each trait using SuSiE. In the scatter plot, one set of genetically colocalized SNPs between CarPlaq (blue) and SIS (red) are identified as triangles marked by black circles. Enlarged points represent fine-mapped SNPs for each trait. Below the scatter plot, the middle panel displays a line plot showing the genes and region associated with each SNP. At the bottom, the triangular heatmap shows the LD structure for the surrounding region. Source data are provided as a Source Data file.

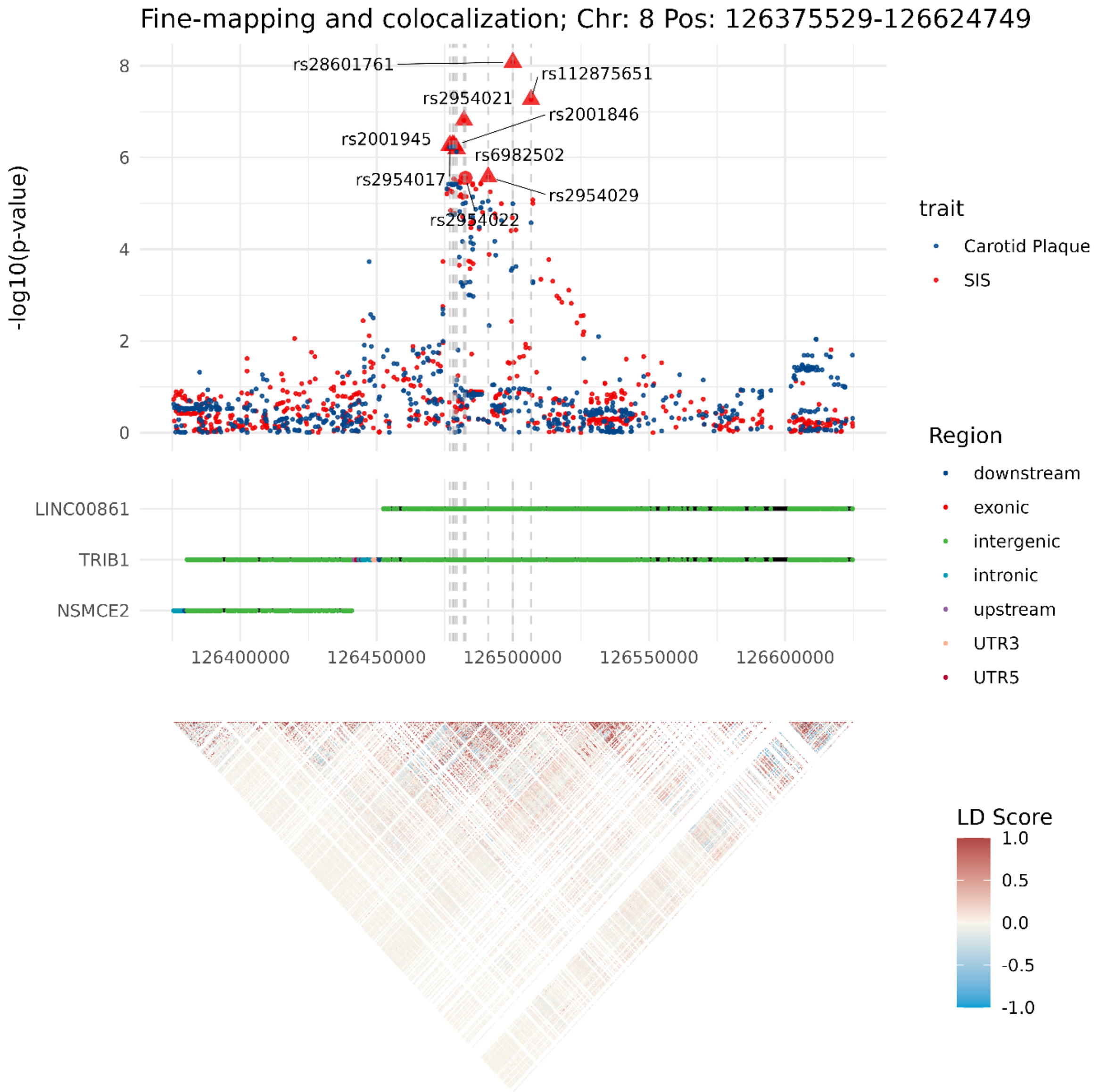

Supplementary Figure S6. Scatter plot of the multivariant colocalization between SIS and CarPlaq for rs4977575.

Genetic variants within a window of 250kb in the locus near rs4977575 were fine-mapped for each trait using SuSiE. In the scatter plot, one set of genetically colocalized SNPs between CarPlaq (blue) and SIS (red) are identified as triangles marked by black circles. Enlarged points represent fine-mapped SNPs for each trait. Below the scatter plot, the middle panel displays a line plot showing the genes and region associated with each SNP. At the bottom, the triangular heatmap shows the LD structure for the surrounding region. Source data are provided as a Source Data file.

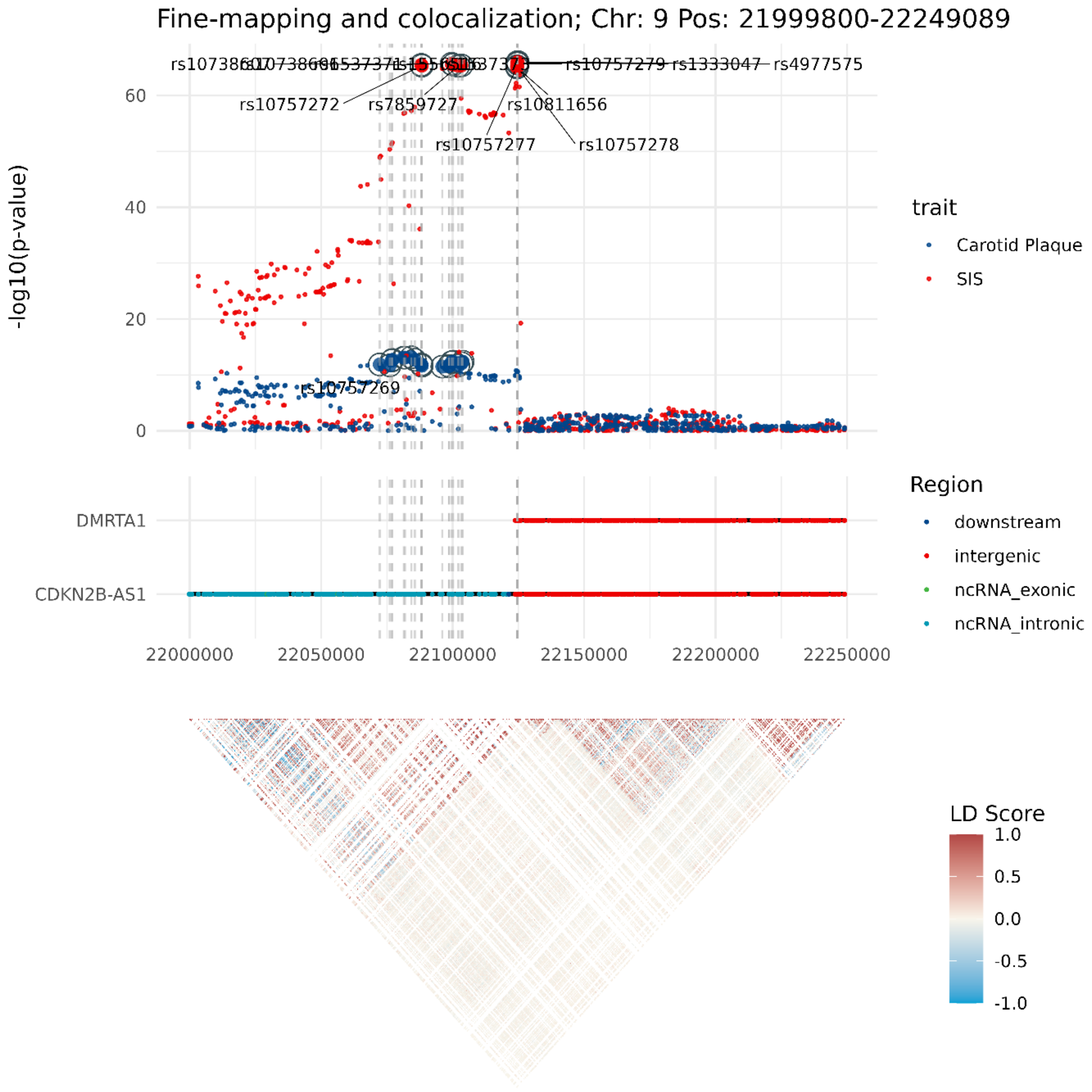

Supplementary Figure S7. Scatter plot of the multivariant colocalization between SIS and CarPlaq for rs7412.

Genetic variants within a window of 250kb in the locus near rs7412 were fine-mapped for each trait using SuSiE. In the scatter plot, one set of genetically colocalized SNPs between CarPlaq (blue) and SIS (red) are identified as triangles marked by black circles. Enlarged points represent fine-mapped SNPs for each trait. Below the scatter plot, the middle panel displays a line plot showing the genes and region associated with each SNP. At the bottom, the triangular heatmap shows the LD structure for the surrounding region. Source data are provided as a Source Data file.

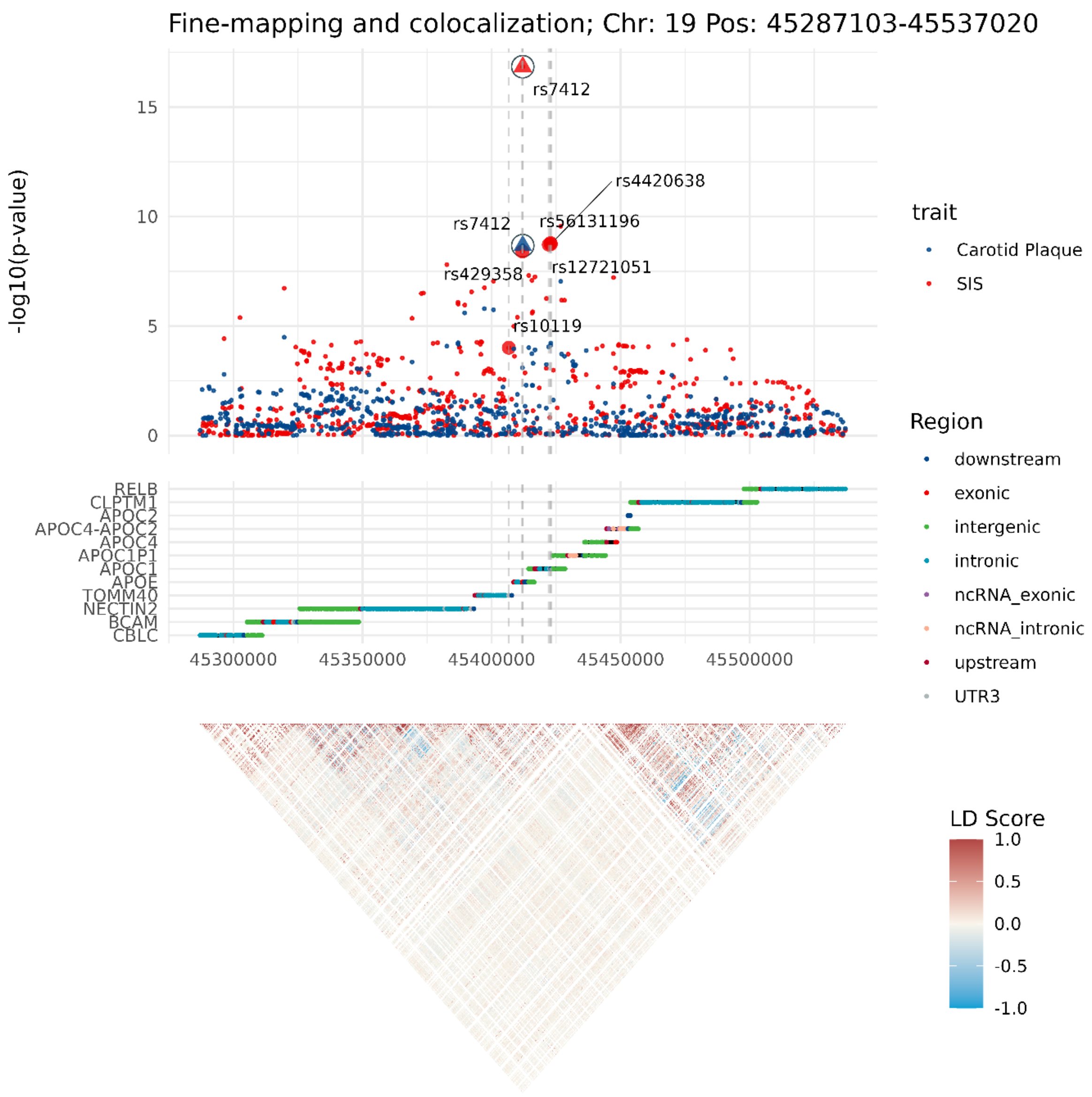

Supplementary Figure S8. Scatter plot of the multivariant colocalization between CarPlaq and SIS for rs1537370.

Genetic variants within a window of 250kb in the locus near rs1537370 were fine-mapped for each trait using SuSiE. In the scatter plot, one set of genetically colocalized SNPs between CarPlaq (blue) and SIS (red) are identified as triangles marked by black circles. Enlarged points represent fine-mapped SNPs for each trait. Below the scatter plot, the middle panel displays a line plot showing the genes and region associated with each SNP. At the bottom, the triangular heatmap shows the LD structure for the surrounding region. Source data are provided as a Source Data file.

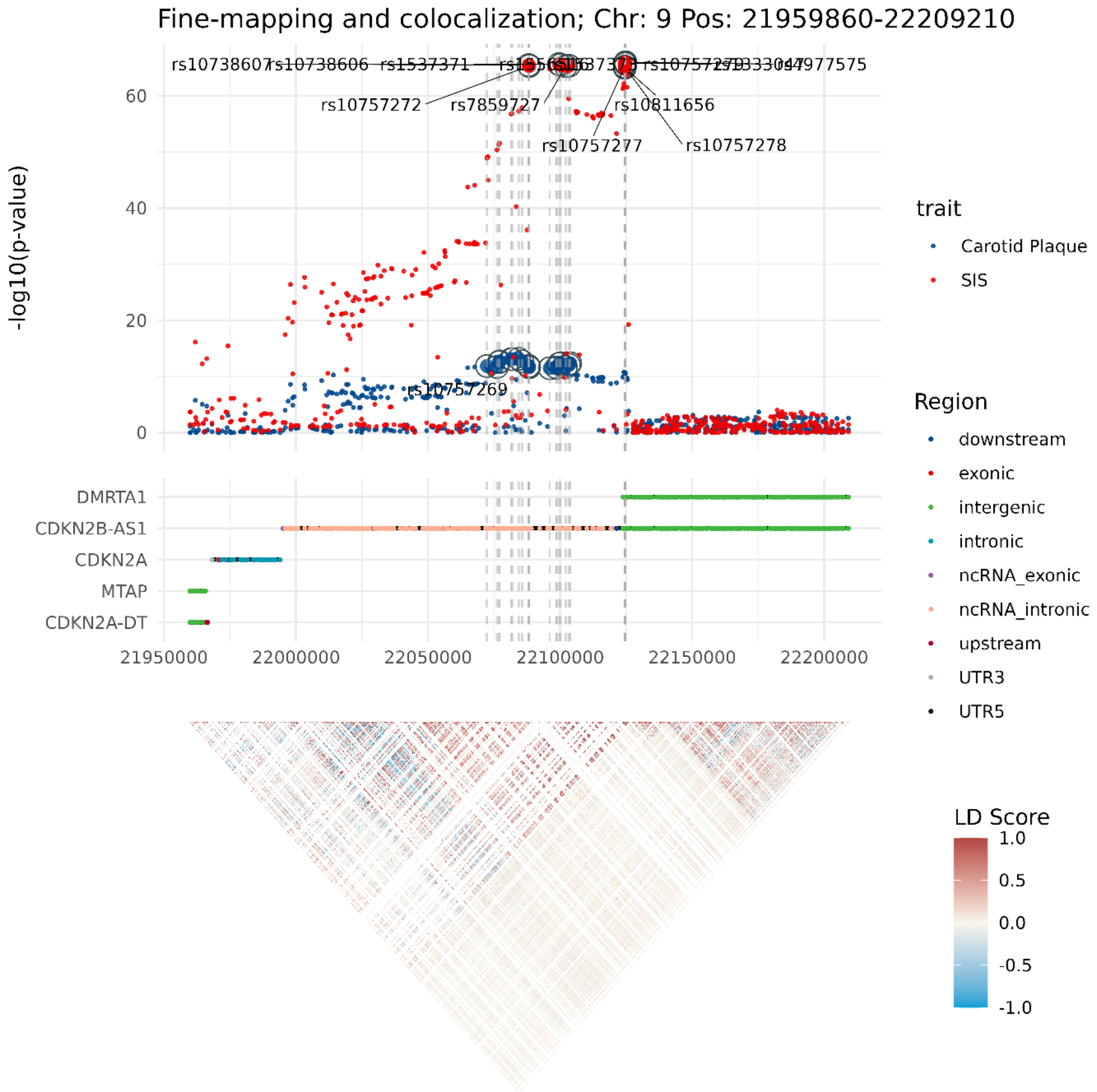

Supplementary Figure S9. Scatter plot of the multivariant colocalization between CarPlaq and SIS for rs7412.

Genetic variants within a window of 250kb in the locus near rs7412 were fine-mapped for each trait using SuSiE. In the scatter plot, one set of genetically colocalized SNPs between CarPlaq (blue) and SIS (red) are identified as triangles marked by black circles. Enlarged points represent fine-mapped SNPs for each trait. Below the scatter plot, the middle panel displays a line plot showing the genes and region associated with each SNP. At the bottom, the triangular heatmap shows the LD structure for the surrounding region. Source data are provided as a Source Data file.

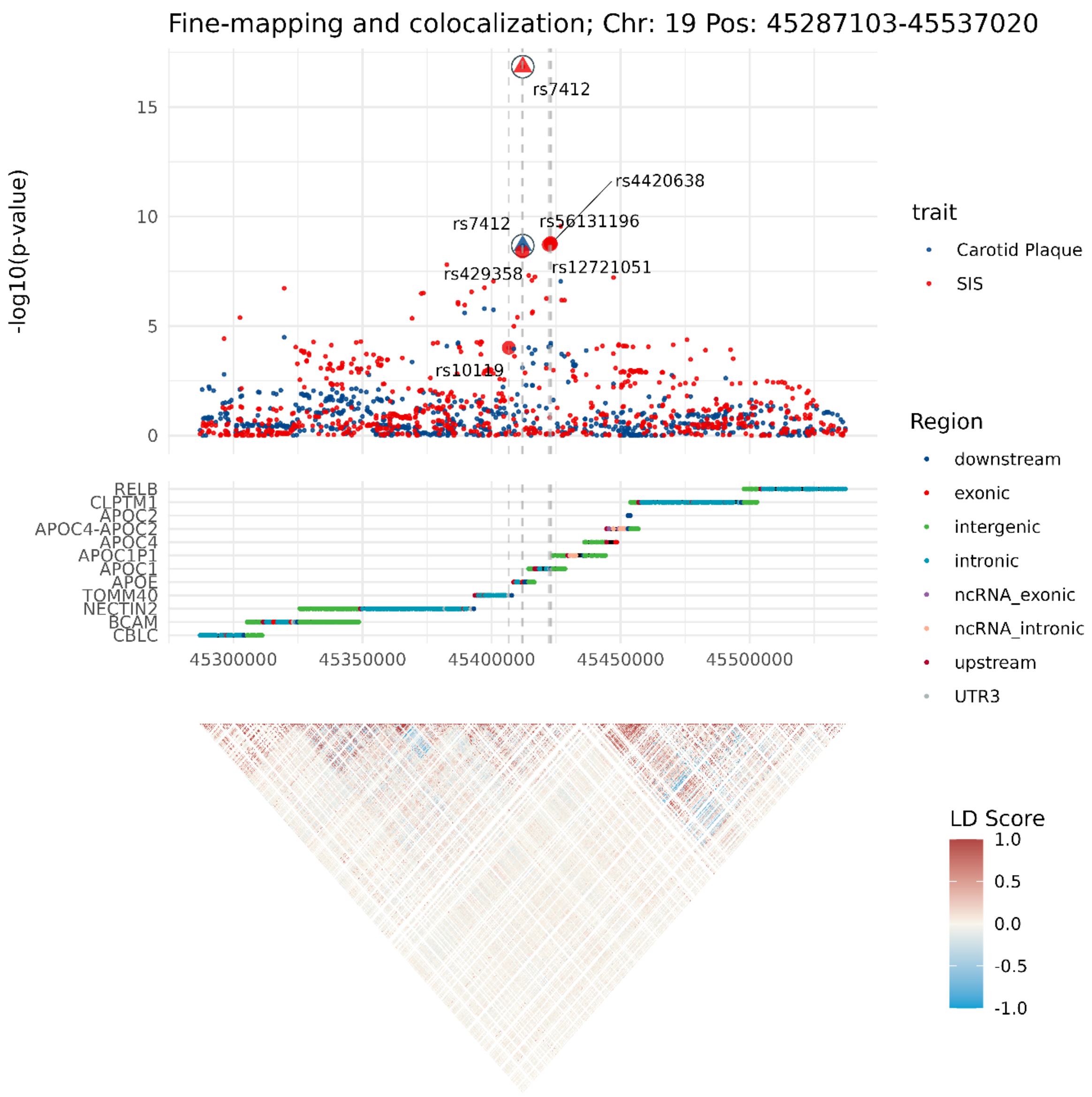

Supplementary Figure S10. Scatter plot of the multivariant colocalization between CarPlaq and SIS for rs74617384.

Genetic variants within a window of 250kb in the locus near rs74617384 were fine-mapped for each trait using SuSiE. In the scatter plot, one set of genetically colocalized SNPs between CarPlaq (blue) and SIS (red) are identified as triangles marked by black circles. Enlarged points represent fine-mapped SNPs for each trait. Below the scatter plot, the middle panel displays a line plot showing the genes and region associated with each SNP. At the bottom, the triangular heatmap shows the LD structure for the surrounding region. Source data are provided as a Source Data file.

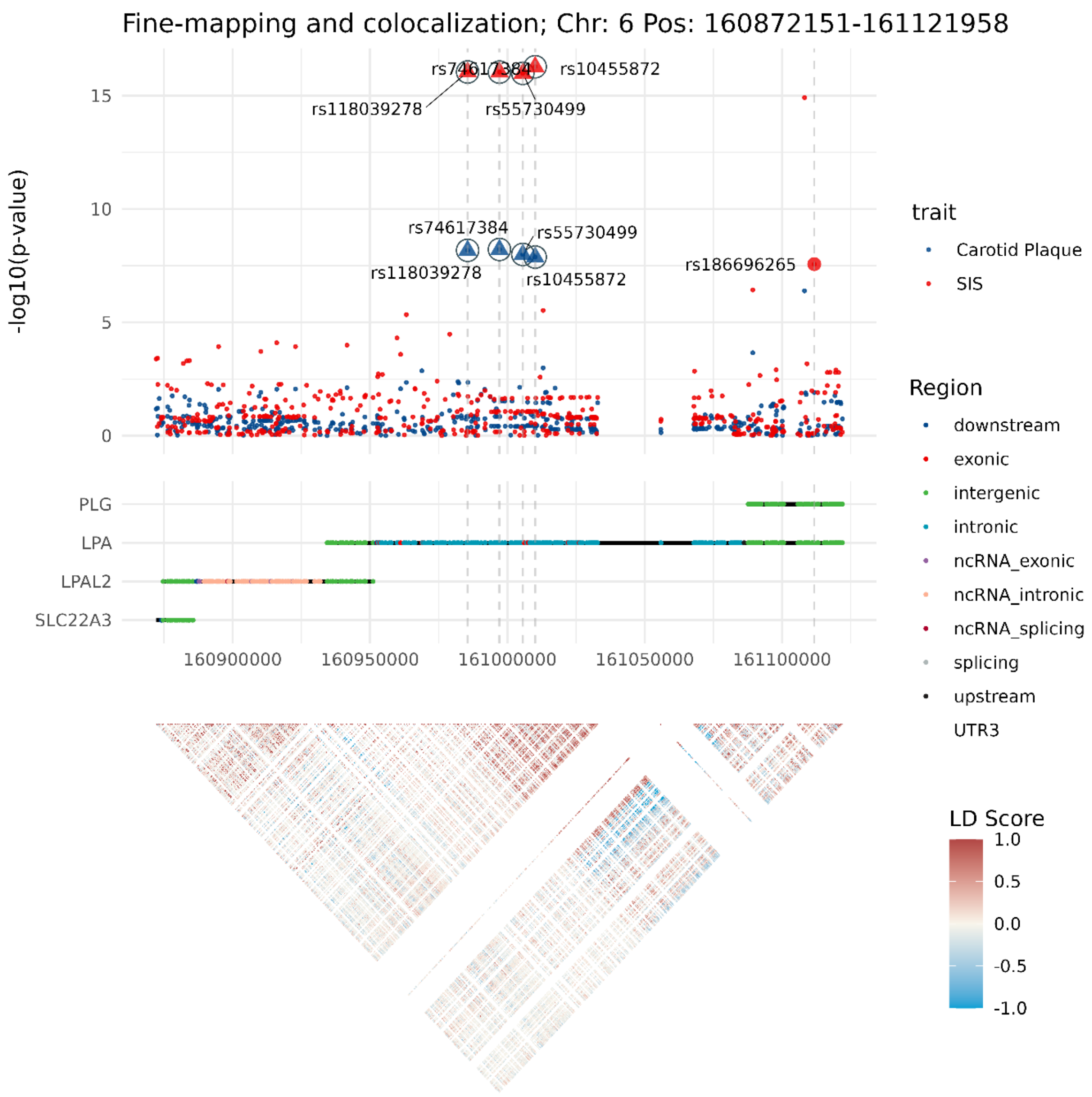

Supplement: Supplementary file 1 — Supplementary Information [file 41467_2025_57457_MOESM1_ESM.pdf]
