# Supplementary material for: A genome-wide association study of imaging-defined atherosclerosis
Source: Nat Commun. 2025 Mar 31;16:2266. doi: 10.1038/s41467-025-57457-7 (PMC11958696; doi:10.1038/s41467-025-57457-7)
Supplement: Supplementary file 2 — Description of Additional Supplementary Files [file 41467_2025_57457_MOESM2_ESM.pdf]

Description of Additional Supplementary Files

File Name: Supplementary Data 1
Description: Genetic associations of significant SNPs vs. coronary artery disease, coronary artery calcification, and carotid intima-media thickness in three recent GWAS publications.
