# Supplementary material for: A genome-wide association study of imaging-defined atherosclerosis
Source: Nat Commun. 2025 Mar 31;16:2266. doi: 10.1038/s41467-025-57457-7 (PMC11958696; doi:10.1038/s41467-025-57457-7)
Supplement: Supplementary file 4 — Reporting Summary [file 41467_2025_57457_MOESM4_ESM.pdf]

nature portfolio

Corresponding author(s): Anders Gummesson

Last updated by author(s): Feb 9, 2025

Reporting Summary

Nature Portfolio wishes to improve the reproducibility of the work that we publish. This form provides structure for consistency and transparency in reporting. For further information on Nature Portfolio policies, see our Editorial Policies and the Editorial Policy Checklist.

Statistics

For all statistical analyses, confirm that the following items are present in the figure legend, table legend, main text, or Methods section.

n/a	Confirmed	
☐	☒	The exact sample size (*n*) for each experimental group/condition, given as a discrete number and unit of measurement
☐	☒	A statement on whether measurements were taken from distinct samples or whether the same sample was measured repeatedly
☐	☒	The statistical test(s) used AND whether they are one- or two-sided<br>*Only common tests should be described solely by name; describe more complex techniques in the Methods section.*
☐	☒	A description of all covariates tested
☐	☒	A description of any assumptions or corrections, such as tests of normality and adjustment for multiple comparisons
☐	☒	A full description of the statistical parameters including central tendency (e.g. means) or other basic estimates (e.g. regression coefficient) AND variation (e.g. standard deviation) or associated estimates of uncertainty (e.g. confidence intervals)
☐	☒	For null hypothesis testing, the test statistic (e.g. *F*, *t*, *r*) with confidence intervals, effect sizes, degrees of freedom and *P* value noted<br>*Give P values as exact values whenever suitable.*
☐	☒	For Bayesian analysis, information on the choice of priors and Markov chain Monte Carlo settings
☒	☐	For hierarchical and complex designs, identification of the appropriate level for tests and full reporting of outcomes
☐	☒	Estimates of effect sizes (e.g. Cohen's *d*, Pearson's *r*), indicating how they were calculated

Our web collection on statistics for biologists contains articles on many of the points above.

Software and code

Policy information about availability of computer code

Data collection	Illumina GenomeStudio 2.0.3 was used to by the genotyping center to call sample genotypes. Genotypes were then imputed at the Sanger Imputation Service with their pipeline utilizing Eagle v2.4 and PBWT v3.1.
Data analysis	Filtering of genotype data: PLINK v2.00a4LM<br>GWAS for ordinal outcome variables: POLMM implementation in the GRAB 0.0.3.6 R-package in R v4.1.1.<br>GWAS continuous outcome variables: Regenie v3.2.5.<br>Definition of independent SNPs: GCTA COJO v1.94.1<br>Colocalization: coloc v5.2.3<br>Genetic correlations: ldsc v1.01<br>Mendelian randomization: MendelianRandomization v0.10.0

For manuscripts utilizing custom algorithms or software that are central to the research but not yet described in published literature, software must be made available to editors and reviewers. We strongly encourage code deposition in a community repository (e.g. GitHub). See the Nature Portfolio guidelines for submitting code & software for further information.

Data

Policy information about availability of data

All manuscripts must include a data availability statement. This statement should provide the following information, where applicable:

- Accession codes, unique identifiers, or web links for publicly available datasets
- A description of any restrictions on data availability
- For clinical datasets or third party data, please ensure that the statement adheres to our policy

The GWAS summary data for imaging-defined atherosclerosis have been deposited in the GWAS Catalog database under the following accession codes: GCST90503075 for SIS (https://www.ebi.ac.uk/gwas/studies/GCST90503075), GCST90503074 for CACS (https://www.ebi.ac.uk/gwas/studies/GCST90503074), and GCST90503076 for CarPlaq (https://www.ebi.ac.uk/gwas/studies/GCST90503076). SCAPIS data are not publicly available due to privacy and ethical restrictions. Access to pseudonymized SCAPIS phenotype and genotype data requires ethical approval from the Swedish Ethical Review Board and approval from the SCAPIS Data access board (https://www.scapis.org/data-access/).

Research involving human participants, their data, or biological material

Policy information about studies with human participants or human data. See also policy information about sex, gender (identity/presentation), and sexual orientation and race, ethnicity and racism.

Reporting on sex and gender	Sex was included as a covariate in the analyses.<br>SCAPIS is a population-based study and sex/gender was not an entry criterion. Information on biological sex was obtained from questionnaires, i.e. self-reported, but also assigned from the genotype data. Removal of mismatches (sex discordance) is part of the standard quality control (QC) of genotype data, hence self-reported and genotyped sex is fully concordant after QC. Data on gender has not been collected in SCAPIS. A sex stratified analysis was not performed since the aim was to identify genetic variants associated with image-based measures of atherosclerosis, and a sex-stratified analysis in this population with relatively small sample size and mild phenotypes would have been underpowered. We do not have consent to report and share individual-level data.
Reporting on race, ethnicity, or other socially relevant groupings	The study includes subjects of European ancestry. No further classification was conducted based on race/ethnicity.
Population characteristics	SCAPIS is a population-based observational cohort with 30,154 subjects aged 50-64 years, randomly recruited from the Swedish population. Population characteristic of the 27,010 subjects (13,110 men and 13,900 women) from SCAPIS with genotypic information are described in Supplementary Table S1. In this subpopulation, the mean age was 57.6 years, mean BMI 26.9 kg/m2. 23.0 % had hypertension, 7.0% had a diabetes diagnosis, and 7.9% had lipid-lowering medication. Coronary artery atherosclerosis, as defined by CCTA, was detected in 42.7% of the subjects.
Recruitment	Between 2013 and 2018, 30,154 subjects aged 50-64 were randomly recruited from the national population register in six areas adjacent to six Swedish university hospitals. No exclusion criteria were applied except the inability to understand written and spoken Swedish for informed consent.
Ethics oversight	This research complies with all relevant ethical regulations. SCAPIS was approved by the Ethical Review Board at Umeå University, Sweden (Dnr 2010-228-31M, Dnr 2017-183-31M, and Dnr 2021-00371). The genetics analysis in SCAPIS was approved by the Swedish Ethical Review Authority (Dnr 2020-04923). All study participants provided informed written consent before enrolment in the study. The participants did not receive compensation for participation.

Note that full information on the approval of the study protocol must also be provided in the manuscript.

Field-specific reporting

Please select the one below that is the best fit for your research. If you are not sure, read the appropriate sections before making your selection.

☒ Life sciences ☐ Behavioural & social sciences ☐ Ecological, evolutionary & environmental sciences

For a reference copy of the document with all sections, see nature.com/documents/nr-reporting-summary-flat.pdf

Life sciences study design

All studies must disclose on these points even when the disclosure is negative.

Sample size	Sample size calculation not applicable for this genome-wide association study, all available data was used.
Data exclusions	Subjects of non-European ancestry were excluded to avoid confounding by population stratification.
Replication	Replication sample not available.
Randomization	Observational data, no randomization.

Blinding	No intervention, hence no blinding.

Reporting for specific materials, systems and methods

We require information from authors about some types of materials, experimental systems and methods used in many studies. Here, indicate whether each material, system or method listed is relevant to your study. If you are not sure if a list item applies to your research, read the appropriate section before selecting a response.

Materials & experimental systems

n/a	Involved in the study
☒	☐ Antibodies
☒	☐ Eukaryotic cell lines
☒	☐ Palaeontology and archaeology
☒	☐ Animals and other organisms
☐	☒ Clinical data
☒	☐ Dual use research of concern
☒	☐ Plants

Methods

n/a	Involved in the study
☒	☐ ChIP-seq
☒	☐ Flow cytometry
☒	☐ MRI-based neuroimaging

Clinical data

Policy information about clinical studies

All manuscripts should comply with the ICMJE guidelines for publication of clinical research and a completed CONSORT checklist must be included with all submissions.

Clinical trial registration	SCAPIS is a observational cohort not registered in ClinicalTrials.org.
Study protocol	Published in Bergstrom, G. et al. The Swedish CArdioPulmonary BioImage Study: objectives and design. J Intern Med 278, 645-59 (2015).
Data collection	Between 2013 and 2018, 30,154 subjects aged 50-64 were randomly recruited from the national population register in six areas adjacent to six Swedish university hospitals.
Outcomes	Primary outcome was coronary atherosclerosis as defined by the segment involvement score from coronary computed tomography angiography. Secondary outcomes were coronary artery calcification score and carotid plaques.

Plants

Seed stocks	*Report on the source of all seed stocks or other plant material used. If applicable, state the seed stock centre and catalogue number. If plant specimens were collected from the field, describe the collection location, date and sampling procedures.*
Novel plant genotypes	*Describe the methods by which all novel plant genotypes were produced. This includes those generated by transgenic approaches, gene editing, chemical/radiation-based mutagenesis and hybridization. For transgenic lines, describe the transformation method, the number of independent lines analyzed and the generation upon which experiments were performed. For gene-edited lines, describe the editor used, the endogenous sequence targeted for editing, the targeting guide RNA sequence (if applicable) and how the editor was applied.*
Authentication	*Describe any authentication procedures for each seed stock used or novel genotype generated. Describe any experiments used to assess the effect of a mutation and, where applicable, how potential secondary effects (e.g. second site T-DNA insertions, mosiacism, off-target gene editing) were examined.*
